# The role of computational and subjective features in emotional body expressions

**DOI:** 10.1038/s41598-020-63125-1

**Published:** 2020-04-10

**Authors:** Marta Poyo Solanas, Maarten J. Vaessen, Beatrice de Gelder

**Affiliations:** 1 Department of Cognitive Neuroscience, Faculty of Psychology and Neuroscience, Maastricht University, Maastricht, 6200 MD The Netherlands; 2 Department of Computer Science, University College London, London, WC1E 6BT UK

**Keywords:** Computational neuroscience, Emotion

## Abstract

Humans are experts at recognizing intent and emotion from other people’s body movements; however, the underlying mechanisms are poorly understood. Here, we computed quantitative features of body posture and kinematics and acquired behavioural ratings of these feature descriptors to investigate their role in affective whole-body movement perception. Representational similarity analyses and classification regression trees were used to investigate the relation of emotional categories to both the computed features and behavioural ratings. Overall, postural rather than kinematic features discriminated better between emotional movements for the computed as well as for the behavioural features. In particular, limb angles and symmetry appeared to be the most relevant ones. This was observed independently of whether or not the time-related information was preserved in the computed features. Interestingly, the behavioural ratings showed a clearer distinction between affective movements than the computed counterparts. Finally, the perceived directionality of the movement (i.e. towards or away from the observer) was found to be critical for the recognition of fear and anger.

## Introduction

Social species spend considerable time watching each others’ body postures and movements since this information is highly relevant for their own behaviour. Often, our understanding of body expressions is direct and automatic as, for example, when we react to an aggressive posture by stepping back. Other times, we are consciously aware of the feelings triggered by the body postures and movements (e.g. fear, anger, dominance)^1,2^. However, neither the body features driving the perception of the emotional content nor the features that play a prominent role in our conscious feelings have been systematically investigated. A better understanding of the core features of nonverbal communication will have a crucial impact on the theories of social interaction, and will directly benefit many areas of society, especially health care, where this knowledge could be useful in the treatment of affective communication disorders.

Most studies have so far investigated how bodies convey emotion by relating verbal descriptions of posture and movement properties to qualitative emotion categories^3,4^. For example, important postural features in discriminating between affective states have been found including head inclination, which is typical for sadness, or limb flexion, which observers associate with the expression of anger^4,5^. Other candidates are the degree of lateral opening of the body (e.g. the body is more extended for happy than for sad), the vertical extension of the body (e.g. hands are often raised for happy but remain low for sad), symmetry (e.g. joy is often depicted by symmetric up and down hand movement) or the directionality of the movement (e.g. forward whole-body movement depicts anger) (for a review see ref. ^6^).

In contrast to the use of qualitative descriptions and categories, computer scientists are increasingly interested in modelling the properties of body postures and movements^6–11^. This requires a detailed analysis of the complex information conveyed by body movements: kinematic (e.g. velocity), dynamics (e.g. mass and force) and posture/form information and its changes over time^12^. For example, with regard to kinematics, it has been found that velocity, acceleration, and jerkiness strongly influence the perception of emotion in expressive arm^13–15^ and also in whole-body movements^10^.

However, the majority of studies investigating the contribution of form and motion information to emotional attribution employed point-light displays (PLD)^16^. While the use of PLDs allows for the control of possible confounds in emotional recognition, such as identity and gender, as well as permitting systematic variations of kinematic and postural features, they are far from representing natural stimuli. Dance movements have also been used^11,17^ but, although more naturalistic, they are often exaggerated and do not represent day-to-day emotional movements, actions or social interactions. The use of static body pictures, on the other hand, obviates the dynamic nature of affective body signals. Therefore, more naturalistic dynamic stimuli are needed to gain insight on how low-level visual body attributes contribute to the perception of specific affective states. Their use, however, comes with difficulties since the configuration of whole-body expressions presents a high dimensionality, and its overall shape varies strongly during movement^10,18^.

Given the inconclusive literature on this topic and the diverse nature of the stimuli used in previous studies, the relative role of postural and kinematic information is not clear. The goal of the present study was to test the hypothesis that kinematic and postural properties reflect differently the affective content of the body movement. To this aim, we pioneered a quantitative representation of naturalistic whole-body movements using computational features and related them to emotion. Our second question concerned the relation of behavioural features derived from measures of subjective perception to the computed features as well as to emotional categories.

## Results

### Computational features

Our first question was whether (dis)similarities in the kinematic and postural features of body movements would reflect the affective categorical structure. For this purpose, several quantitative body features were computed from affective body-movement videos expressing anger, fear, happiness or a non-emotional expression. To compute the features, we estimated the position of the actors’ main joints using the state-of-the-art 2D pose estimation library OpenPose (v1.0.1)^19^. Kinematic features included velocity, acceleration and vertical movement (i.e. amount of displacement of each *keypoint* in the y-axes between adjacent frames). Postural features consisted of symmetry (i.e. distance between each pair of joints with respect to the axis that divides the body vertically by the nose), limb angles (i.e. angle between two adjacent body segments, including the angles for the elbows, knees, shoulders and hips) and three different computations of body contraction: shoulder ratio (i.e. amount of extension of the body joints with respect to the shoulders), surface (i.e. area spanned by the total body extension in the x-axis and the extension in the y-axis) and limb contraction (i.e. average of the distances between the wrists and ankles to the head).

**Figure 1:**
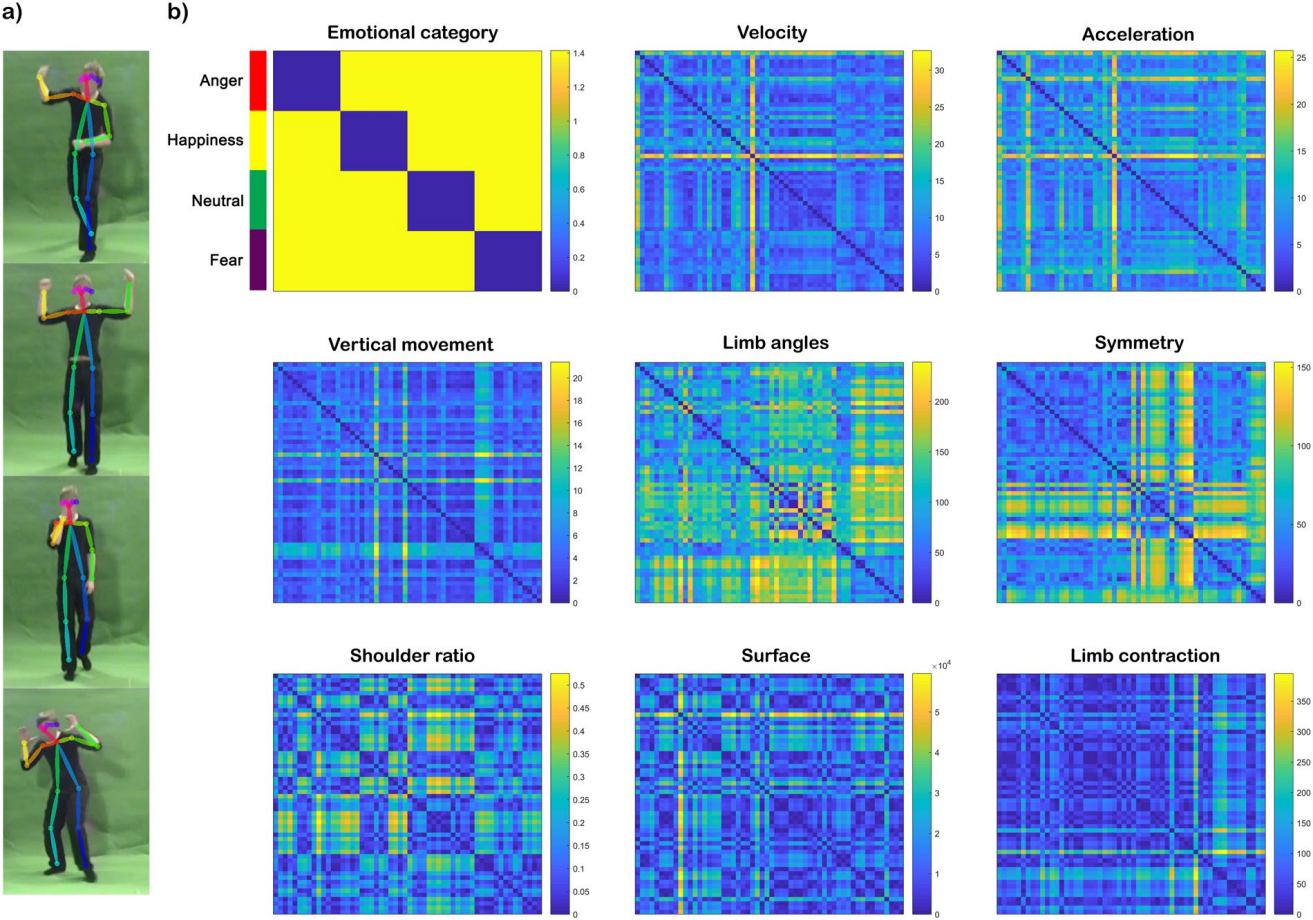
Representational dissimilarity matrices of the kinematic and postural features. (**a**) Example of a key video-frame per emotional category from our stimulus set with the OpenPose skeleton overlaid on top. From top to bottom: angry, happy, neutral and fearful stimuli. (**b)** The RDMs represent pairwise comparisons between 56 stimuli with regard to the kinematic (i.e. velocity, acceleration and vertical movement) and postural (i.e. limb angles, symmetry, shoulder ratio, surface and limb contraction) computed features averaged over time (see Supplementary Materials for more information). The dissimilarity measure reflects Euclidean distance, with blue indicating strong similarity and yellow strong dissimilarity. Colour lines in the upper left corner indicate the organization of the RDMs with respect to the emotional category (anger: red; happiness: yellow; neutral: green; fear: purple) of the video stimuli.

To investigate the relationship between these features and emotion categories we used representational similarity analysis (RSA)^20,21^. This method is based on the computation of the relations between pairs of stimuli, which are represented in the so-called representational dissimilarity matrices (RDMs). These matrices are, therefore, able to capture the level of (dis)similarity across stimuli and allow the comparison between different stimuli representations and data modalities (see Materials and methods). Figure 1 illustrates the results of the representational similarity analysis conducted with the computed features. The resulting RDMs were arranged in the same order as the four-emotional categories RDM (see Fig. 1b, upper left corner). The (dis)similarity structure of symmetry showed a clear dissociation between neutral and the rest of the affective body movements. Likewise, the neutral condition presented more similarities to itself with respect to shoulder ratio and limb angles than to the other emotional classes. For limb angles, the fearful condition also showed a high degree of within-category similarity and between-category dissimilarity. Kinematic RDMs such as the ones for velocity, acceleration and vertical movement did not reflect a clear differentiation between emotional categories.

**Figure 2:**
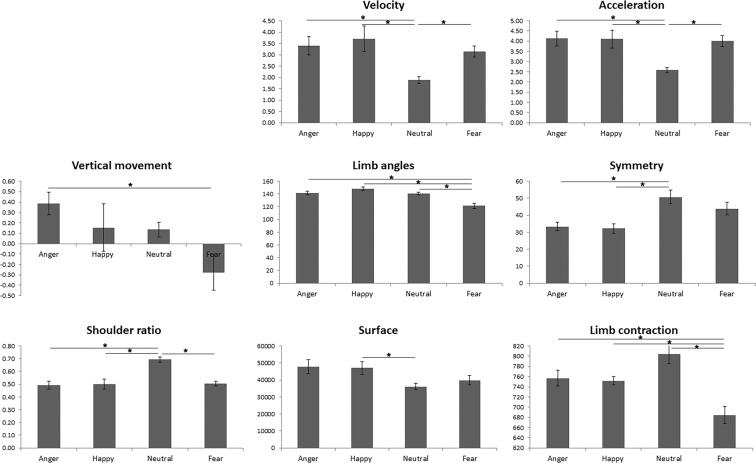
Feature differences across emotions. For each feature, an ANOVA with a four-level factor Emotion (Anger, Happiness, Neutral and Fear) was computed using each video’s averaged feature values as input. Lines and asterisks indicate Bonferroni-corrected significant pairwise comparisons (p < 0.05).

To investigate whether the observed differences between emotional categories were significant, a one-way repeated-measures ANOVA was computed, separately, for each feature (see Fig. 2). Velocity showed a significant main effect of emotion (F(1.764, 22.926) = 4.835, p = 0.021, η_p_^2^ = 0.271), with Bonferroni corrected post-hoc pairwise comparisons showing that the angry (M = 3.4, t(13) = 3.946, p_Bonf_ = 0.01), happy (M = 3.71, t(13) = 3.109, p_Bonf_. = 0.05) and fearful (M = 3.14, t(13) = 4.903, p_Bonf_ = 0.002) conditions significantly faster than the neutral one (M = 1.9). Acceleration also presented a main effect of emotion (F(3, 39) = 5.202, p = 0.004, η_p_^2^ = 0.286), with anger (M = 4.13, t(13) = 4.364, p_Bonf_ = 0.004), happiness (M = 4.11, t(13) = 3.126, p_Bonf_ = 0.048) and fear (M = 4.01, t(13) = 4.753, p_Bonf_ = 0.002) showing significantly higher acceleration values than neutral movements (M = 2.58). A significant main effect of emotion was also observed in the case of vertical movement (F(3,39) = 3.226, p = 0.033, η_p_^2^ = 0.199), where angry expressions (M = 0.39, t(13) = 3.114, p_Bonf_ = 0.048) presented more vertical displacement than fearful ones (M = −0.28). Limb angles showed a significant main efect of emotion (F(3,39) = 13.499, p < 0.001, η_p_^2^ = 0.509), with the limbs in the angry (M = 141.87, t(13) = 3.376, p_Bonf_ = 0.03), happy (M = 148.36, t(13) = 6.381, p_Bonf_ < 0.001) and neutral (M = 140.39, t(13) = 4.719, p_Bonf_ = 0.002) conditions being significantly less flexed than in fear (M = 121.27). The main effect of emotion was significant in the case of symmetry (F(3,39) = 7.372, p < 0.001, η_p_^2^ = 0.362), with angry (M = 33.27, t(13) = −3.651, p_Bonf_ = 0.018) and happy (M = 32.26, t(13) = −3.463, p_Bonf_ = 0.025) movements being significantly less symmetrical than neutral ones (M = 50.68). The significant main effect of emotion in shoulder ratio (F(3,39) = 17.416, p < 0.001, η_p_^2^ = 0.573) revealed that angry (M = 0.49, t(13) = −8.084, p_Bonf_ < 0.001), happy (M = 0.5, t(13) = −5.21, p_Bonf_ = 0.001) and fearful bodies (M = 0.51, t(13) = −7.847, p_Bonf_ < 0.001) were significantly more extended than neutral ones (M = 0.69). Surface presented a significant main effect of emotion (F(3,39) = 3.712, p = 0.019, η_p_^2^ = 0.22), with happy (M = 47054.6, t(13) = 3.379, p_Bonf_ = 0.03) being significantly more extended than neutral movements (M = 36090.46). Finally, the significant main effect of emotion for limb contraction (F(3,39) = 10.410, p < 0.001, η_p_^2^ = 0.445) revealed that angry (M = 756.99, t(13) = 3.455, p_Bonf_ = 0.026), happy (M = 751.89, t(13) = 4.542, p_Bonf_ = 0.003) and neutral bodies (M = 803.6, t(13) = 4.787, p_Bonf_ < 0.001) were significantly more extended than the fearful ones (M = 684.63).

**Figure 3:**
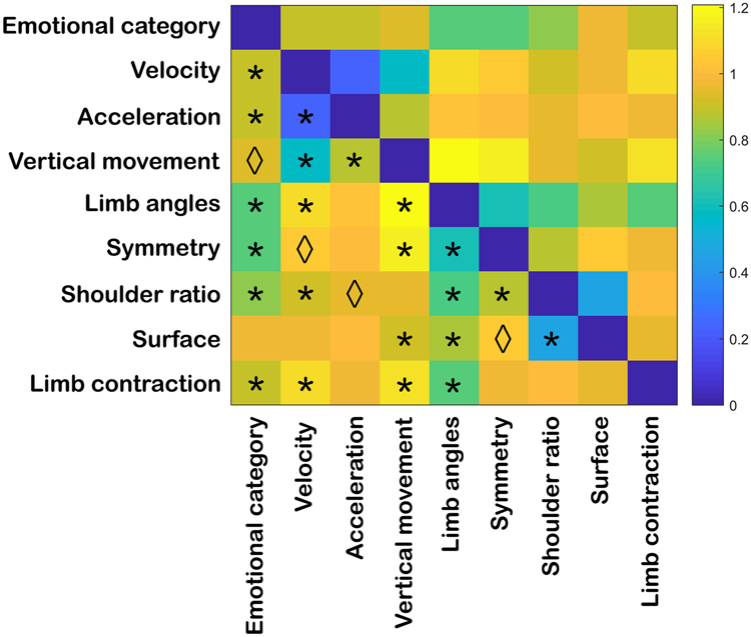
Correlation between representational dissimilarity matrices of kinematic and postural features. The RDM represents the level of (dis)similarity between each of the kinematic (i.e. velocity, acceleration and vertical movement) and postural (i.e. limb angles, symmetry, shoulder ratio, surface and limb contraction) matrices (see Fig. 1). Distances are indicated in 1-Spearman’s correlation values, with blue indicating strong similarity and yellow strong dissimilarity. Asterisks and rhombi below the diagonal indicate significant correlations after Bonferroni correction and correlations that presented significant uncorrected p-values, respectively (α_bonf_ = 0.05/9, with nine comparisons per feature; see Table SR1 in Supplementary Results for correlation and p-values).

To examine whether the kinematic and postural attributes relate to each other and/or to the emotional categories, pairwise comparisons were computed between the corresponding matrices (Fig. 3; see Table SR1 in Supplementary Results for correlation and p-values; below only p-values corrected for multiple comparisons are reported). Interestingly, both postural and kinematic RDMs were positively correlated, although weakly, with the emotion RDM with the exception of vertical movement and surface: velocity (r(1538) = 0.094, p = 0.002), acceleration (r(1538) = 0.101, p = 0.001), limb angles (r(1538) = 0.251, p < 0.001), symmetry (r(1538) = 0.262, p < 0.001), shoulder ratio (r(1538) = 0.185, p < 0.001), and limb contraction (r(1538) = 0.112, p < 0.001). The feature matrices that most strongly correlated with the emotion RDM were, therefore, limb angles and symmetry. Kinematic RDMs overall correlated more strongly among each other, specially velocity and acceleration (r(1538) = 0.768, p < 0.001) while postural matrices showed weaker correlations. Among these, limb angles and symmetry (r(1538) = 0.377, p < 0.001), and shoulder ratio and surface (r(1538) = 0.547, p < 0.001) presented the relatively strongest correlations. The relationship between postural and kinematic matrices was weak and often negative.

**Figure 4:**
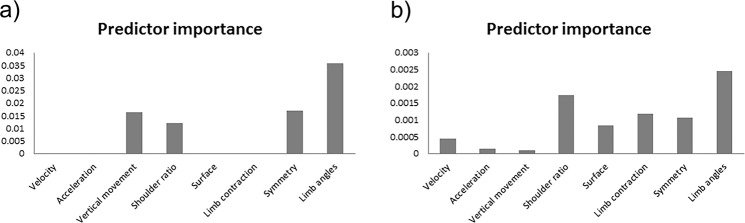
Feature importance for the classification of emotion. Two decision tree classifiers were trained and tested with the eight computed features as predictors and the four emotional categories as the predicted class. Kinematic features included velocity, acceleration and vertical movement. Postural features included limb angles, symmetry, shoulder ratio, surface and limb contraction. (**a)** Predictor relevance for the classification model where the postural and kinematic features were averaged over time (overall classification accuracy of 61%, with chance level at 25%); (**b)** Predictor relevance for the classification model where the postural and kinematic features kept the temporal information (overall classification accuracy of 84%, with chance level at 25%).

To further investigate the relationship between affective states and kinematic and postural attributes of body movement, two decision tree classifiers were trained and tested. The eight computed features were used as predictors and the emotional categories as the predicted class. The two models differed in whether or not the feature descriptors kept the temporal information of the movement. The model using features averaged over time provided an overall classification accuracy of 61% (angry = 35%, happy = 62%, neutral = 63%, fear = 95%; see Fig. SR1A in Supplementary Results for more details on classification accuracy by emotion) and showed that the angles between the limbs, symmetry, and the vertical displacement of the body joints are the most relevant features for the classification of emotion from body movements (see Fig. 4a**;** see Fig. SR2 in Supplementary Results for an overview of the classification tree). When using feature descriptors that kept the temporal information (e.g. using information from individual frames), limb angles still appeared as the most relevant predictor, together with shoulder ratio and limb contraction (see Fig. 4b). Importantly, this second model gave the higher accuracy of 84% (angry = 79%, happy = 83%, neutral = 92%, fear = 83%; see Fig. SR1B in Supplementary Results for more details on classification accuracy by emotion).

Two more decision trees were performed that investigated whether a specific body part was most responsible for the recognition of emotion. This analysis revealed that the left side of the body was more relevant than the right side or the head/nose position in the distinction between emotions (classification accuracy> 90%, see Fig. SR3A in Supplementary Results). A more detailed examination showed that the wrists, especially the left one, were the most important body parts in the classification of affect (classification accuracy> 95%, see Fig. SR3B in Supplementary Results).

### Behavioural ratings

**Figure 5:**
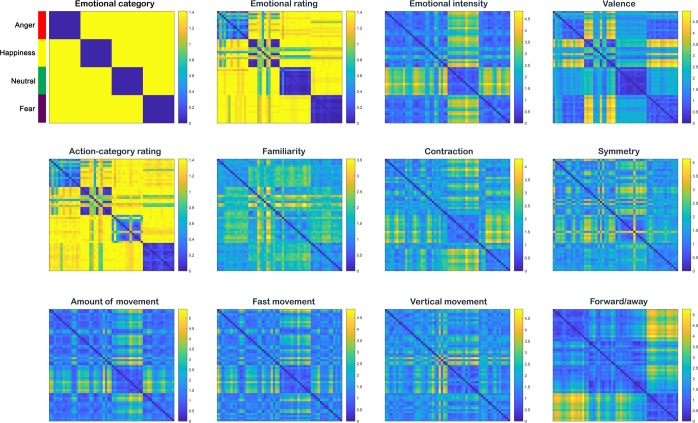
Representational dissimilarity matrices of the behavioural ratings. The RDMs represent pairwise comparisons between the 56 stimuli with regard to the each of the behavioural ratings (see Supplementary Materials for more information). The dissimilarity measure reflects Euclidean distance, with blue indicating strong similarity and yellow strong dissimilarity. Colour lines in the upper left corner indicate the organization of the RDMs with respect to the emotional category (anger: red; happiness: yellow; neutral: green; fear: purple) of the video stimuli.

A further goal of this study was to investigate the (dis)similarity of different emotional body movements with regard to the perceived kinematic and postural features. For this purpose, 30 participants answered six questions concerning kinematic (i.e. amount of movement, fast movement, vertical movement, direction of the movement) and postural (i.e. body contraction, symmetry) aspects of the movement. To gain more insight on their perception of the stimuli, five more questions were asked about emotional- (i.e. emotional category, intensity, familiarity, valence) and action-related traits (i.e. action category) of the stimuli (see Methods and Supplementary Materials for more information on the behavioural task). The assessment of the level of inter-rater agreement revealed a high consistency (> 90%) across participants in the all the ratings (see Supplementary Table SR2). Figure 5 shows the average perceptual (dis)similarity scores across participants for each of the possible combinations of the 56 videos, for each rating, respectively. The (dis)similarity structure of kinematic-related ratings, such as amount of movement, fast and vertical movement, showed a relatively clear dissociation between neutral and the rest of the affective body movements. Likewise, the ratings for the neutral condition presented more within-category similarity with respect to contraction and symmetry than to the other emotional classes. This distinction was also clearly marked in the emotional intensity matrix, where the neutral category presented high within-category similarity and between-category dissimilarity. The four emotional categories did not show differences with regard to emotional intensity. However, participants reported having different degree of familiarity across categories: anger, neutral and fear presented within-category consistence while this was not the case for happiness. With respect to between-category comparisons for familiarity, only anger and fear showed some degree of similarity, whereas dissimilarity dominated the rest of the comparisons. A high within-category similarity was observed for valence. As could be expected, happiness displayed a strong dissimilarity to fear and anger with respect to valence while the latter ones presented higher similarity. For the forward/away rating, the fearful and angry conditions showed a high degree of within-category similarity and between-category dissimilarity while the other categories were more similar within and between each other.

**Figure 6:**
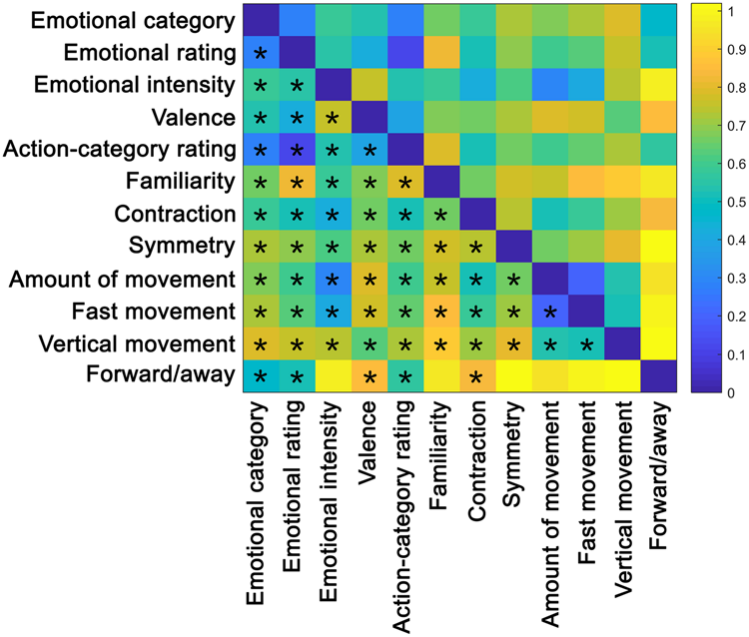
Correlation between representational dissimilarity matrices of the different behavioural ratings. The RDM represents the level of (dis)similarity between each of the behavioural-rating matrices (see Fig. 5). Distances are indicated in 1-Spearman’s correlation values, with blue indicating strong similarity and yellow strong dissimilarity. Asterisks below the diagonal indicate significant correlations after Bonferroni correction (α_bonf_ = 0.05/12, with 12 comparisons per behavioural rating; see Table SR3 in Supplementary Results for correlation and p-values).

To examine whether the kinematic-, postural-, emotional- and action-related ratings correlate to each other and/or to the emotional categories, pairwise comparisons were computed between the corresponding matrices (see Fig. 6 and Table SR3 in Supplementary Results for correlation and p-values; below only p-values corrected for multiple comparisons are reported). The behavioural rating on emotional categories correlated positively with all the behavioural ratings, showing the strongest correlations with valence (r(1538) = 0.580, p < 0.001) and action-category ratings (r(1538) = 0.887, p < 0.001). Participant’s emotional ratings correlated positively with the emotional categories was well (r(1538) = 0.728, p < 0.001). Indeed, participants classified the affective body movements with high accuracy (see Fig. SR4 in Supplementary Results for an inspection of the confusion matrix, also Fig. 5 for the perceptual similarity RDM with respect to emotion). Neutral and fear were the most accurately recognised categories with 97 and 98% correct classification rate, respectively, while movements intended to express happiness had the lowest correct emotion attribution (78%), being most often confused with neutral body movements. In addition, the emotional categories correlated positively with all the remaining behavioural ratings, the strongest correlations being with action-category ratings (r(1538) = 0.720, p < 0.001) and forward/away (r(1538) = 0.535, p < 0.001). As with the computed features, kinematic ratings correlated more strongly among each other while postural ratings exhibited weaker correlations. The comparison between postural and kinematic ratings showed overall moderate to weak positive correlations.

**Figure 7:**
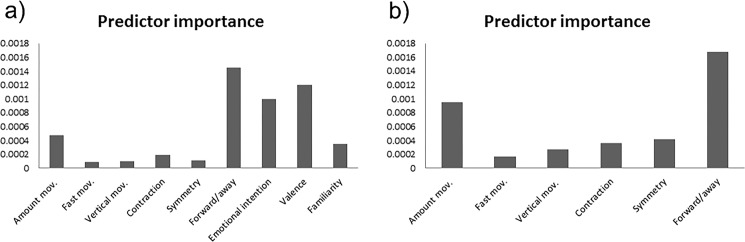
Behavioural rating importance for the classification of emotion. (a) Predictor relevance for the classification model where the ratings of postural (i.e. contraction and symmetry), kinematic (i.e. amount of movement, fast movement, vertical movement and forward/away) and emotional (i.e. emotional intention, valence and familiarity) traits were included (overall classification accuracy of 78%, with chance level at 25%); (**b)** Predictor relevance for the classification model where only postural and kinematic ratings were included (overall classification accuracy of 71%, with chance level at 25%).

To further investigate possible contributions of the perceived kinematic, postural and emotional attributes to the classification of emotion, two decision tree classifiers were trained and tested. The classification of emotion was performed using either (1) all behavioural ratings excluding emotional category rating and action category rating or (2) only the six behavioural ratings that represented kinematic or postural aspects of the movement (i.e. excluding emotional category, action category, emotional intensity, valence, and familiarity ratings). The first model gave a classification accuracy of 78% (angry = 73%, happy = 68%, neutral = 85%, fear = 87%; see Fig. SR5A in Supplementary Results for more details on classification accuracy by emotion) and showed that the ratings of forward/away, valence and emotional intensity are the most relevant descriptors for the classification of emotional body movements (see Fig. 7a). When using only the behavioural ratings that presented computed counterparts, forward-away was again the most relevant descriptor, followed by amount of movement and symmetry (see Fig. 7b). This second model gave the lower accuracy of 71% (angry = 64%, happy = 58%, neutral = 76%, fear = 84%; see Fig. SR5B in Supplementary Results for more details on classification accuracy by emotion).

### Comparison between computational and perceived body features

**Figure 8:**
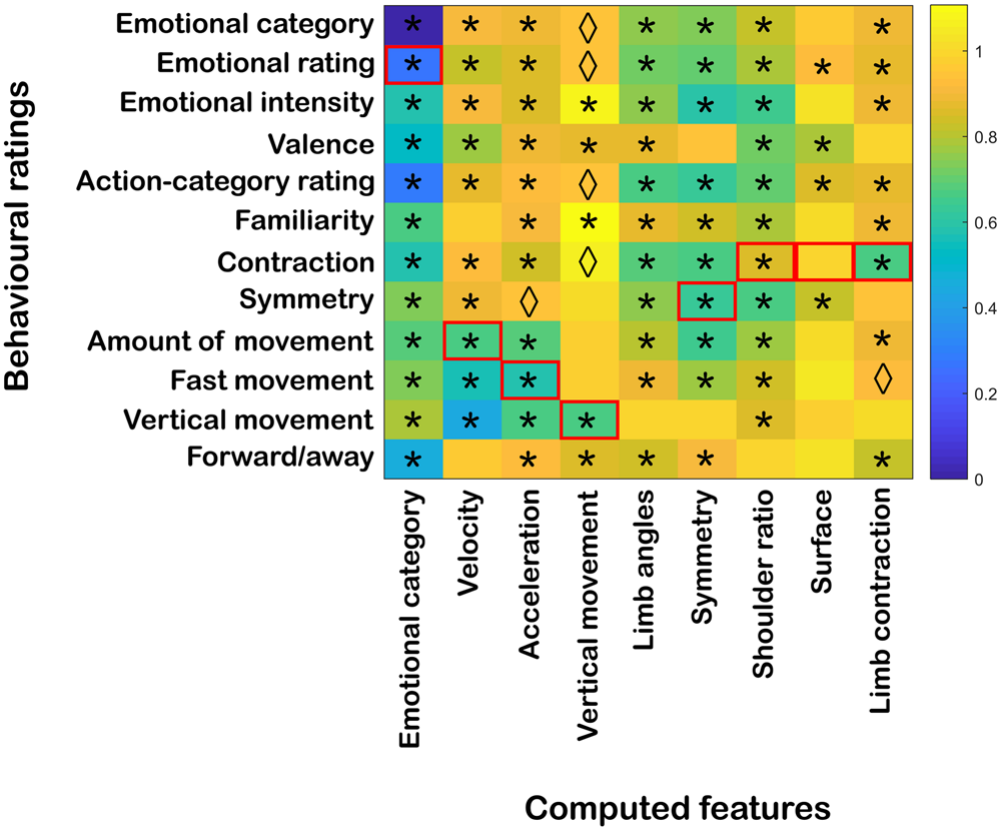
Average Spearman’s rank correlation between the behavioural-rating RDMs and the RDMs of the computed features. Distances are indicated in 1-Spearman’s correlation values, with blue indicating strong similarity and yellow strong dissimilarity between behavioural and computed features. Asterisks and rhombi below the diagonal indicate significant correlations after Bonferroni correction and correlations that presented significant uncorrected p-values, respectively (α_bonf_ = 0.05/12, with 12 comparisons per behavioural rating; see Table SR4 in Supplementary Results). Red boxes indicate the correspondence between computed features and behavioural ratings.

This study also aimed at investigating whether perceptual (dis)similarities in kinematic and postural attributes across videos could be predicted by corresponding (dis)similarities at the computational level. To investigate this relationship, each of the perceptual RDMs was correlated to every computed feature RDM (Fig. 8**;** see Table SR4 in Supplementary Results for correlation and p-values). With regard to the behavioural assessments (see previous section), the matrices showing the strongest positive correlation to emotion categories belonged to emotional ratings, action categories and forward/away. In the case of the computed features, the strongest positive correlations to emotion categories were found for postural features such as symmetry, limb angles and shoulder ratio (see Fig. 3).

The behavioural task was designed such that the ratings would reflect common usage and still correspond to some of the computed features (see Fig. 8, where these correspondences are indicated as red squares). When evaluating the actual correlations between the behavioural and computed attributes at the RDM level, some of the behavioural ratings were indeed correlated the highest to their computational counterpart, although not in all cases. The behavioural rating of symmetry indeed showed the highest correlation with the computed symmetry. However, the behavioural rating of contraction (which conceptually is related to the computation features shoulder ratio, surface and limb contraction) correlated highest with symmetry. The ratings of amount of movement, fast movement and vertical movement, which are conceptually related to computed velocity, acceleration and vertical movement, respectively, were all correlated significantly between each other. However, the behavioural rating of amount of movement was also highly correlated with symmetry, whereas fast movement correlated the strongest with velocity. Also, the rating of vertical movement was actually more strongly correlated to velocity than its computed counterpart.

## Discussion

This study used an innovative approach to investigate the role of kinematic and postural information in whole-body movement perception. Quantitative features of posture and movement derived from the position of the actors’ main joints were related to emotion categories as well as to behavioural ratings of feature descriptors. Overall, postural rather than kinematic features seemed to discriminate better between different emotional body movements, both for the computed features as well as for the behavioural ratings. Among the postural descriptors, limb angles and symmetry appeared to be the most important cues. Moreover, adding time-related information to the computed features significantly improved the classification accuracy as well as changed the contribution of specific features in emotion classification. Finally, the perceived directionality of the movement (i.e. towards or away from the observer) was found to be critical for the recognition of fear and anger.

### Computational features

Our first RSA result indicates that, overall, postural and not kinematic computational features differentiated best between emotional categories (see Fig. 1). This finding was further supported by the correlation analyses between emotion and feature RDMs and the decision tree classifiers, where the postural features of limb angles and symmetry correlated best with emotional category (see Fig. 3) and were the two most important features for the classification of emotion (see Fig. 4a). These findings are in agreement with previous literature suggesting that postural cues are the critical features for the discrimination of emotion while motion cues only provide additional information used to solve occasional ambiguity^12,22–24^. For example, Atkinson and colleagues (2007) found that although movement cues were sufficient for affective recognition, the disruption of form information severely impaired the recognition performance. More support to this hypothesis comes from the neuropsychological study by McLeod (1996). In this study, a brain-damaged patient was still able to detect human actions using solely form cues from motion despite presenting a deficit in perceiving moving stimuli. It has also been found that biological motion-selective areas are more sensitive to spatial than temporal scrambling of PLD configurations^25^. Our finding, thus, underscores that postural rather than kinematic features play a more important role in the mechanism of decoding emotion from body movements.

Interestingly, the relevance of postural descriptors over kinematic ones was not only observed when removing the time information of the movement (i.e. averaging feature values over time) but even when preserving this information (see Fig. SR6 in Supplementary Results). The classification accuracy was also higher for the model whose feature descriptors preserved the time information, although using time-averaged features still provided above-chance classification performance. This suggests that time information may not be strictly necessary to distinguish between emotions, but provides additional information that can be used to solve difficult cases^23^. This finding also concurs with the importance of form over motion information in the classification of emotion found in previous literature (for a review see ref. ^6^). Yet, the way in which these features were computed seems to be critical in determining the relevance of some attributes over others. Without the time information, limb angles, symmetry and the vertical displacement of the joints were the most relevant features for emotion classification. Keeping the time information showed that limb angles still appeared as the most relevant predictor, but together with shoulder ratio and limb contraction.

Previous studies have also shown that different body expressions are best described by different contributions of postural and kinematic features^3,4^. For example, it has been revealed that postural cues have a stronger influence compared to dynamic cues in anger^26^ and fear expressions^12,27^ than for happiness or sadness. In line with this finding, we found that limb angles and limb contraction are relevant in differentiating fear from other expressive movements (see Fig. 1 and Fig. 2). The amount of vertical displacement seems, however, important in discerning fear from anger. Interestingly, velocity, acceleration and shoulder ratio play a role in differentiating emotional from non-emotional body movements. These results may not be apparent from the representational similarity analyses in the case of velocity and acceleration. A possible explanation could be that while the former used time- and keypoint-averaged data, the latter only time-averaged information.

### Behavioural features

Our second result concerns the behavioural features and how they relate to emotion categories. This provides a picture of which features may predominantly guide subjective emotion recognition, yet not providing a direct link. Here, the examination of participants’ perceptual judgements about kinematic as well as contraction and symmetry descriptors revealed a clear discrimination between neutral and the rest of the affective categories (see Fig. 5). Interestingly, the perceived directionality of the movement (i.e. forward/away) also seemed to be relevant for the classification of emotion (see Fig. 7), especially for the discrimination between fear and anger (see Fig. 5). This may explain the high recognition rate observed for fearful expressions, and that fearful expressions were rarely confused with angry ones, and vice versa (see Fig. SR4 in Supplementary Results). This is in line with previous literature showing that avoidance behaviour is a diagnostic feature of fear^12,28^, while approaching behaviour is for anger^28^. For example, Hortensius and colleagues (2016) found that the recognition accuracy of fearful body expressions was higher when the movement was directed away rather than towards the observer whereas the opposite pattern was observed for angry expressions. Interestingly, the authors reported an increase in the excitability of the motor cortex for angry expressions regardless of the directionality of the movement, while motor cortex excitability was not affected by the directionality of fearful expressions^28^. These findings are in agreement with the idea of an evolutionary link between action and emotion for adaptive behaviour^29^. In the presence of a threatening signal, the directionality of the movement may help the observer to prepare for an adaptive action.

### Relation between computational and behavioural features

As mentioned in previous sections, the distinction between different affective movements using computed kinematic features was unclear. However, participants’ ratings on the corresponding attributes revealed a relatively clearer distinction between emotional and non-emotional movements (see Fig. 5). This gap between computed and perceptual descriptors was also reflected in the weak correlations between their respective matrices (see Fig. 8). It could be that the approach followed to calculate the kinematic features does not characterize well how people process movement information for the distinction between different affective movements.

With regard to postural cues, more similarities were found between the computed and perceptual features. The clearest example was observed with symmetry. In the case of the behavioural rating of “contraction”, three different computed counterparts were defined: shoulder ratio, surface and limb contraction. However, the behavioural rating of this attribute was closer to the representation of limb contraction and limb angles than to surface or shoulder ratio. As this example shows, a specific attribute can be computed in indeed multiple ways.

### Body parts and the expression of emotion

It is known from previous studies that different body parts play different roles in the distinction between emotions^4,13,30,31^. For example, there is evidence of pronounced lateral asymmetries in the way the body conveys emotion. Specifically, it has been found that the left side of the body is more emotionally expressive than the right^31^. Similarly, some authors have shown that the movement of the upper body only^30^ or even of a single arm^13,15^ or hand^4^ may be enough to distinguish between affective states. In line with these findings, the current study also found that the left side of the body is more relevant for the classification of emotion (see Fig. SR3A in Supplementary Results). A more detailed investigation revealed that the wrists, especially the left one, are the most important body parts in the classification of affect (see Fig. SR3B in Supplementary Results). In view of the previous literature, these results point to a property of the emotional body expression rather than to an artefact of stimuli recording or analysis.

### Methodological advancement, limitations and future directions

An important difficulty in studying body movement and posture is the fact that the body is a complex high-dimensional stimulus^10^. At present, there are still very few studies investigating emotion expression at the level of the whole body. Previous studies going beyond the face have often selected a body part that most significantly contributed to the distinction between emotions^4,13,30,31^. Taking a more methodological and systematic approach, other authors investigating affective movements, but also intention and motor control, have used data-reduction methods such as PCA^32–34^, factor analysis^35–38^ or blind-source separation algorithms^10^. Our study is the first to create a quantitative description of naturalistic whole-body movements using postural and kinematic features selected for their demonstrated importance in previous literature (for a review see ref. ^6^.). This approach was adopted since relevant body descriptors obtained from perceptual experiments have been shown to be in good agreement with those extracted from data-driven approaches^10^. This was only possible due to the new developments in machine learning algorithms for person detection (e.g. OpenPose^19^). The selection of a body part would have reduced the dimensionality of the defined features, but our aim was to pioneer the investigation of how whole-body movements, rather than its parts, convey emotion. The definition of the core features of human movement not only allowed us to investigate their relation to emotional categories, but importantly to also establish the link to observers’ subjective perception of feature descriptors.

Nevertheless, it is important to be aware of the limitations of our findings. For instance, although the actors in the current study were coached to express affective expressions in a naturalistic way, futures studies could also explore real-life emotional expressions. Future studies may also use larger and more diverse stimulus sets with a wider range of affective states, also covering multimodal, contextual and cross-cultural matters. In addition, we tested male and female participants but the actors in our video clips were always male. The assessment of the inter-rater reliability revealed a high consistency across participants (see Table SR2 in Supplementary Results) and no significant differences between female and male participants were found for the behavioural RDMs with the exception of “fast movement”. These findings suggest that the unbalanced gender stimuli selection is not crucial for the interpretation of our findings. Nevertheless, future studies should circumvent this possible confound. Finally, OpenPose occasionally lead to inaccuracies in joint position estimation that had to be manually corrected. These inaccuracies were most frequent when a body part was occluded. Future studies may benefit from other methodologies, such as the use of MoCap data, to circumvent this issue.

A next step is the further understanding of these features in relation to the different brain regions involved in body perception. Insights in these mechanisms will have a crucial impact on our understanding of affect and social interaction, but also in many areas of society, including law enforcement and security, games and entertainment, education, the arts^17^ but, most importantly, health care^6^. Patients suffering from disorders of affective communication, such as autism and schizophrenia, will directly benefit from the application of this knowledge to rehabilitation programmes focused on emotional recognition and normal social functioning.

## Materials and methods

### Participants

Thirty-two volunteers participated in the behavioural experiment, but only the data of thirty (mean age = 22.97; age range = 19-36; ten males; four left-handed participants, one of them male) were included in the analysis due to technical issues in data recording. All participants had normal or corrected-to-normal vision and a medical history without any psychiatric or neurological disorders. The experiment was performed in accordance with the Declaration of Helsinki and all procedures followed the regulations of the Ethical Committee at Maastricht University. All participants provided written informed consent before taking part in the experiment. Participants either received credit points or were reimbursed with vouchers.

### Stimuli

Stimuli consisted of 56 one-second video clips (25 frames) of whole-body movements. In each video, a male actor expressed one out of three possible emotional body movements: happy, fearful or angry. The stimulus set also included neutral body actions such as coughing, pulling the nose or walking. Therefore, this experiment consisted of four categories (i.e. happy, fear, anger, neutral), each of them consisting of 14 videos with seven male actor identities.

The stimuli were computer-edited using Ulead, After Effects and Lightworks (EditShare). To avoid triggering facial perception processes, the faces of the actors were blurred with a Gaussian mask so only the information of the body was available. In addition, all actors were dressed in black and filmed against a green background under controlled lighting conditions. The video clips included in this experiment belonged to a larger stimulus set and were selected based on a high recognition accuracy (>80%). For more information regarding the recording and validation of these stimuli, see ref. ^39^.

### Pose estimation

A state-of-the-art 2D skeleton extraction library called OpenPose (v1.0.1)^19^ was used to infer each actor’s pose in the video stimuli. OpenPose uses a convolutional neural network to estimate the position of the main joints in a total of 18 *keypoints* (i.e. ears, eyes, nose, neck, shoulders, elbows, hands, left and right part of the hip, knees and feet). Each *keypoint* is defined by its x and y image coordinates and a confidence value indicative of the algorithm’s certainty in determining the position of the joint. Subsequently, OpenPose uses part affinity fields to associate the *keypoints* in order to produce an anatomically inspired skeleton (see Fig. SM1 Supplementary Materials). Due to the blurring of the face in our video clips, the estimation of the location of eyes and ears was often inaccurate. These *keypoints* were disregarded for further analysis giving that our purpose is the computation of kinematic and postural body features. However, the *keypoint* corresponding to the nose was kept for further analysis as a reference for the position of the head. Thus, three values were obtained for the remaining 14 *keypoints* and 25 frames for each of the 56 videos of the stimulus set. In addition, visual inspection of the estimated joint positions was performed to assess the accuracy of the algorithm and manual corrections were performed when necessary with the help of Adobe Photoshop CS6’s coordinate system (v13.0, Adobe Systems Inc., San Jose, CA, USA).

### Feature definition

To investigate the possible contribution of kinematic and postural body attributes to the processing and recognition of emotional movements, several quantitative features were computed giving their importance in previous work (for a review see ref. ^6^). Kinematic features included velocity, acceleration and vertical movement while postural features consisted of symmetry, limb angles and three different computations of body contraction (i.e. shoulder ratio, surface and limb contraction). These features were calculated using custom code in MATLAB (vR2017a, The MathWorks Inc., Natick, MA, USA) from the x- and y-coordinates of the 14 *keypoints* and 25 frames of each of the 56 videos. Although each feature was calculated within each frame, the time information was later averaged (see Supplementary Materials for more information on feature definition).

### Experimental design, task and procedure

For this behavioural experiment, the 56 videos that comprised the body-movement stimulus set were presented in four runs lasting approximately 15 minutes, respectively. In each run, 14 video stimuli were shown, each repeated 11 times. Each trial consisted of 100 ms fixation period followed by one-second video presentation. Immediately after each video presentation, participants were required to answer one out of 11 questions with regard to kinematic- (i.e. amount of movement, fast movement, vertical movement, direction of the movement), posture- (i.e. body contraction, symmetry), emotion- (i.e. emotional category, intensity, familiarity, valence), and action- (i.e. action category) related aspects of the body movement displayed in the video (see Supplementary Materials). The questions about kinematic and postural aspects were closely related to the computed features, and were rated on a seven-point scale using a computer mouse. The emotional and action categorization questions required a forced-choice answer (see Supplementary Materials). The stimulus presentation order was randomized, both within and between runs, for each participant. However, the experimental questions were performed in the same order, and in consecutive trials, across participants for each video (i.e. first postural and kinematic ratings, followed by more emotional-related traits; see Supplementary Table SM1 in Supplementary Materials) to avoid triggering high-cognitive processes when answering feature-related questions. Therefore, for each participant 11 ratings were obtained for each of the 56 videos. Before the actual experiment, instructions and a practice run were provided to the participants. The stimuli were displayed using PsychoPy2 (v1.90.0)^40,41^ in the centre of a computer screen (screen resolution = 1920 ×1200; screen refresh rate = 60 Hz) under controlled lighting conditions. The stimuli spanned 14.03 degrees of visual angle.

### Assessment of inter-rater reliability

We computed the Intraclass Correlation Coefficient in SPSS to evaluate the level of inter-rater agreement. For this purpose, a two-way random model assessing absolute agreement among participants was computed per behavioural rating.

### Representational similarity analysis

Relations among the computed features and behavioural ratings were calculated by means of representational similarity analyses^20,21^ in MATLAB (vR2017a, The MathWorks Inc., Natick, MA, USA). This approach involves the comparison of pairs of stimuli values to determine their representational dissimilarity. RSA characterizes this representation by means of representational dissimilarity matrices, which are symmetrical. The diagonal entries reflect comparisons between identical stimuli and were defined as zero. Each off-diagonal value indicates the dissimilarity between values associated with two different videos.

#### Computed-feature and behaviour-based RDMs

Based on the computed features for each of the 56 videos, RDMs were constructed by defining a dissimilarity value for all stimulus pairs in Euclidean distance (i.e. first-level feature RSA). For the emotional categories RDM, dummy variables were used such that the same emotion had zero dissimilarity with itself while two different emotions presented a dissimilarity of √2. A first-level RSA was also performed with the behavioural ratings using Euclidean distance. For each rating, a group RDM was produced by averaging the RDMs of all participants. For the emotional and action ratings, dummy variables were used as in the case of the emotional category RDM. This analysis generated 56×56 distance matrices for both the computed features and the behavioural ratings. To examine the relationship between each computed feature and perceptual rating, Spearman’s rank correlations were carried out, resulting in second-level RDMs. Spearman’s correlations were also performed to investigate possible correlations between features and behavioural ratings, respectively.

### Classification regression trees

In order to investigate the relative importance of the kinematic and postural features as well as the participant’s ratings in the classification of affective body movements, decision tree classifiers^42^ were implemented in the Machine Learning Toolbox (v11.1) from MATLAB (vR2017a, The MathWorks Inc., Natick, MA, USA). The classification was achieved by means of binary splits, finding a decision criterion that best separated the multi-class data at each node into two groups. The decision criterion for this binary division was based on the attribute (e.g. feature) that returned the highest information gain. In addition, the classification of the data was based not only on one individual tree but on the weighted majority of multiple decision trees^43^. With this bootstrap-aggregating approach, the effects of overfitting were reduced, improving generalization. The importance of each feature in the classification of the affective body movements was obtained from the tree using the full data.

Four decision trees were considered for the classification of emotion using computed and behavioural ratings, which differed in the predictors used: (1) the postural and kinematic features averaged over time and *keypoints;* (2) the postural and kinematic features averaged over *keypoints* while keeping the temporal information; (3) nine behavioural ratings (i.e. all behavioural ratings excluding the emotional and action category ratings) and (4) only the six behavioural ratings that represented kinematic or postural aspects of the body movement (i.e. excluding the emotional and action category ratings as well as the ratings of emotional intensity, valence and familiarity). These different sets of descriptors were used as input for each decision tree classifier, respectively.

In addition, two more decision trees were performed to investigate whether a given body part most significantly contributed to the distinction between emotions. Specifically, the first tree examined whether there were lateral asymmetries in the way the body expressed affect. This tree used as predictors the average value of the all the keypoint positions at the centre (i.e. nose and neck), at the left (i.e. left shoulder, elbow, wrist, hip, knee and ankle) and right side of the body (i.e. right shoulder, elbow, wrist, hip, knee and ankle), respectively, of each video. The second tree used fourteen descriptors representing the average keypoint location of the fourteen body joints, for all the videos.

### One-way repeated-measures ANOVA

A one-way repeated-measures ANOVA was conducted in SPSS for each feature to investigate possible differences between emotional categories. Each ANOVA was, therefore, constituted of a four-level factor Emotion (i.e. Anger, Happiness, Neutral and Fear) and used as input the feature’s averaged values of each video. In the cases where sphericity was violated, Greenhouse-Geisser correction was applied.

## Supplementary information

Supplementary information.

## Data Availability

The datasets generated and analysed during the current study are available from the corresponding author on reasonable request.
