# Supplementary material for: The role of computational and subjective features in emotional body expressions
Source: Sci Rep. 2020 Apr 10;10:6202. doi: 10.1038/s41598-020-63125-1 (PMC7148324; doi:10.1038/s41598-020-63125-1)
Supplement: Supplementary file 1 — Supplementary information. [file 41598_2020_63125_MOESM1_ESM.pdf]

Supplementary Information for:

The role of computational and subjective features in emotional body expressions

Marta Poyo Solanas[1], Maarten Vaessen[1], & Beatrice de Gelder[1,2,*]

[1] Department of Cognitive Neuroscience, Faculty of Psychology and Neuroscience, Maastricht University, Maastricht, Limburg 6200 MD, The Netherlands.

[2] Department of Computer Science, University College London, London WC1E 6BT, UK.

* Address correspondence to Beatrice de Gelder, Department of Cognitive Neuroscience, Brain and Emotion Laboratory, Faculty of Psychology and Neuroscience, Maastricht University, Oxfordlaan 55, 6229 EV Maastricht, The Netherlands. Tel. +31 433881437. E-mail: b.degelder@maastrichtuniversity.nl

Supplementary Materials

Feature definition

To investigate the contribution of kinematic and postural information to the processing and recognition of emotional body movements, several quantitative features were computed giving their importance in previous work (for a review see REF.[1]). These features include velocity, acceleration, vertical movement, symmetry, limb angles, shoulder ratio, surface and limb contraction. However, there are different ways in which these features can be calculated. Here, a stimulus dataset comprising 56 affective body-movement videos expressing either anger, fear, happiness or a non-emotional expression were used. The x- and y-coordinates of a total of 18 *keypoints* that corresponded to the actor's main body joints were acquired using OpenPose (v1.0.1)[2], for each of the 25 frames that constituted each video (see **Fig. SM1**). Due to the blurring of the face in our video clips, the estimation of the location of eyes and ears was often inaccurate. These *keypoints* were disregarded for further analyses, although the *keypoint* corresponding to the nose was kept as a reference for the position of the head. Velocity was calculated as the amount of displacement of each *keypoint* for adjacent frames. Acceleration was derived from the difference in the amount of movement of each *keypoint* for adjacent frames. Vertical movement referred to the amount of displacement of each *keypoint* in the y-axes between adjacent frames. The feature defined as "limb angles" was calculated as the angle between two adjacent body segments, including the angles for the elbows, knees, shoulders and hips. Symmetry was computed as the difference in position of each pair of joints (i.e. one on the left side, the other on the right) with respect to the axis that divides the body vertically by the nose. Shoulder ratio was defined as the amount of extension of the body joints with respect to the shoulders. Surface was computed as the multiplication between the total body extension in the x-axis and the extension in the y-axis. Limb contraction referred to the feature where the

distances of the limbs (wrists and ankles) to the head were estimated. Although each feature was calculated within each frame, the results presented in the main text correspond to features whose time information was averaged. In the supplementary results, the dissimilarity matrices of the features preserving the time-related information are shown (see **Fig. SR6**). Finally, emotional categories simply denoted whether a video belonged to a category (anger, happiness, neutral or fear) or not.

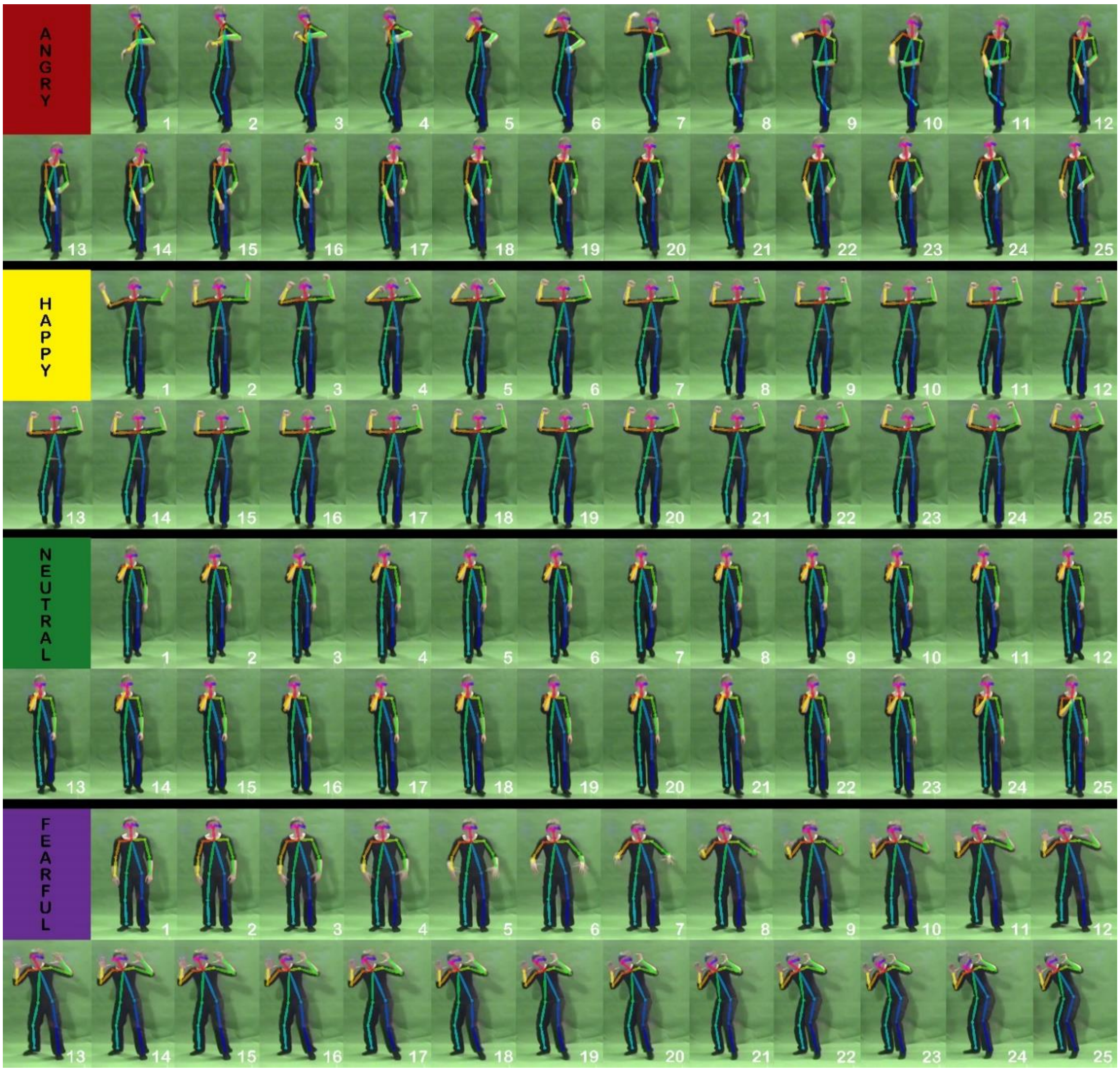

Figure SM1. Example of videos from our stimulus set depicting the different emotional categories with the OpenPose skeleton.

Experimental questions for the behavioural part of the experiment

One of the goals of this study was to investigate the (dis)similarity of different emotional body movements with regard to the perceived kinematic and postural attributes. For this purpose, 30 participants answered six questions concerning kinematic (i.e. amount of movement, fast movement, vertical movement, direction of the movement) and postural (i.e. body contraction, symmetry) aspects of the movement. To gain more insight on their perception of the stimuli, five more questions were asked about emotional- (i.e. emotional category, intensity, familiarity, valence) and action- related (i.e. action category) traits of the stimuli. The movement- and postural- related questions were rated on a seven-point scale, whereas the emotional and action categorization ones were forced-choice. The order and the content of the questions can be seen in **Table SM1**.

Table SM1. Experimental questions and ratings for the behavioural part of the experiment

Questions	Answer
1.- How much body movement is there in the video?	Little movement = 1; A lot of movement = 7
2.- How fast is the body movement in the video?	Very slow = 1; Very fast = 7
3.- How much vertical movement is shown in the video?	Little movement = 1; A lot of movement = 7
4.- How much body contraction is there in the body movement?	Little contraction = 1; A lot of contraction = 7
5.- How symmetrical is the body movement?	Little symmetrical = 1; Very symmetrical = 7
6.- Is the movement directed towards you or away from you?	Away from you = 1; Towards you = 7
7.- Which action do you think is represented by the movement?	Walking, threatening, celebrating, coughing, self-protecting
8.- How familiar is the body movement to you?	Very unfamiliar = 1; Very familiar = 7
9.- What is the valence of the body movement?	Very negative = 1; Very positive = 7
10.- Which emotion do you think the person is expressing?	Anger, happiness, neutral, fear
11.- How intense is the emotion being expressed?	Little intense =1; Very intense = 7

Supplementary Results

Emotional recognition and validity of the stimuli

Participants accurately classified the emotion expressed by the body movements, indicating the validity of the stimuli for the purpose of the study. Fear and neutral conditions were the best recognized, whereas happiness was the worst, being most often confused with neutral body expressions (see **Fig. SR4** in Supplementary Results). The observation that emotional intensity ratings were similar across emotions excludes this factor as a possible confound in the misclassification of happiness (see **Fig. 5** in main text). In addition, the movements conveying this emotion had similar kinematic and postural within-category similarity (see **Fig. 1** in main text). A possible explanation for this selective misclassification could be the different level of familiarity that participants presented with each affective movement. An inspection of the matrix for familiarity ratings (**Fig. 5** in main text) revealed that while fear, neutral and anger categories had high within-category similarity, the happy condition displayed less within-category consistency. Another possible explanation could be the existence of individual differences in affective expression between actors. Indeed, a closer look at participants' emotional ratings indicated that the lower recognition accuracies were specifically attributed to two actors.

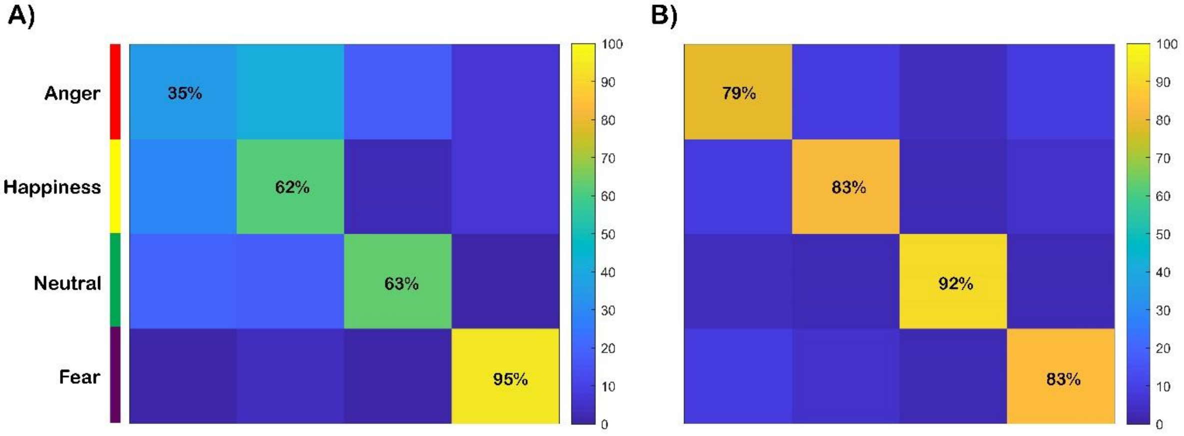

Figure SR1. Confusion matrices showing the classification accuracy by emotional category of the models using computed features. A) Emotion classification accuracy of the model where the postural (i.e. limb angles, symmetry, shoulder ratio, surface and limb contraction) and kinematic (i.e. velocity, acceleration and vertical movement) features were averaged over time (overall classification accuracy of 61%, with chance level at 25%); **B)** Emotion classification accuracy of the model where the postural and kinematic features kept the temporal information (overall classification accuracy of 84%, with chance level at 25%). Colour lines in the left indicate the organization of the matrices with respect to the emotional category (anger: red; happiness: yellow; neutral: green; fear: purple).

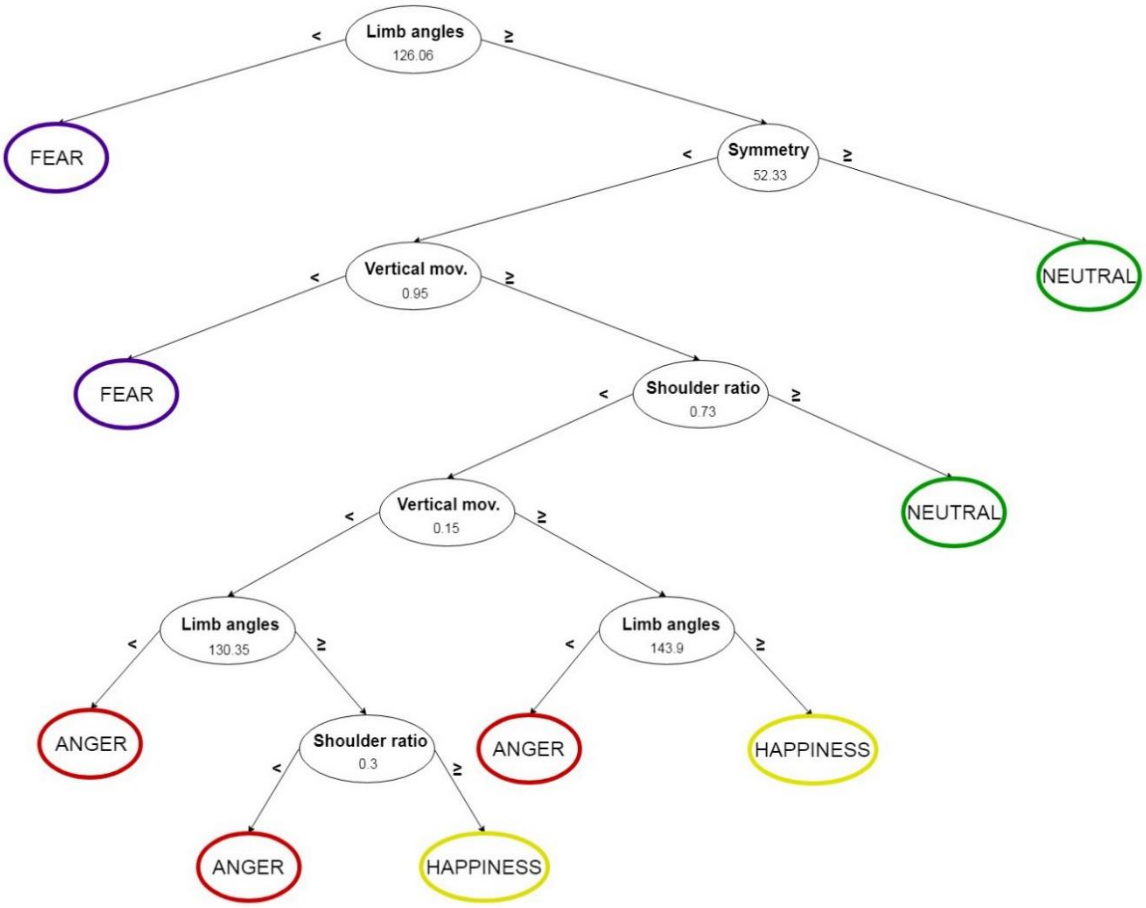

Figure SR2. Emotional classification from postural and kinematic features averaged over time. A decision tree classifier was trained and tested (classification accuracy of 61%, with chance level at 25%) with the eight computed features as predictors and the four emotional categories as the predicted class. Kinematic features: velocity, acceleration and vertical movement. Postural features: limb angles, symmetry, shoulder ratio, surface and limb contraction.

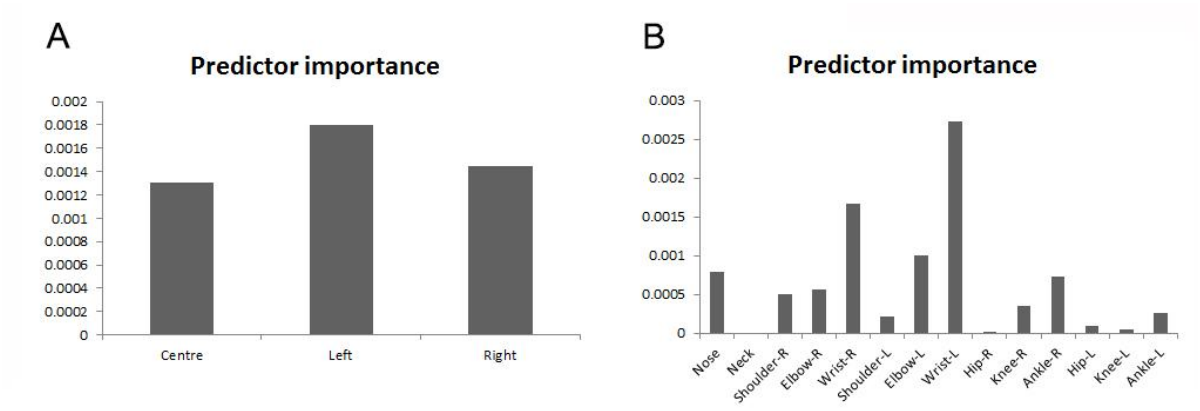

Figure SR3. Body part importance for the classification of emotion. A) Predictor relevance for the classification model using the average of the *keypoints* at the centre of the body (i.e. nose and neck), at the left (i.e. left shoulder, elbow, wrist, hip, knee and ankle) and right side (i.e. right shoulder, elbow, wrist, hip, knee and ankle); **B)** Predictor relevance for the classification model using all the fourteen body *keypoints*.

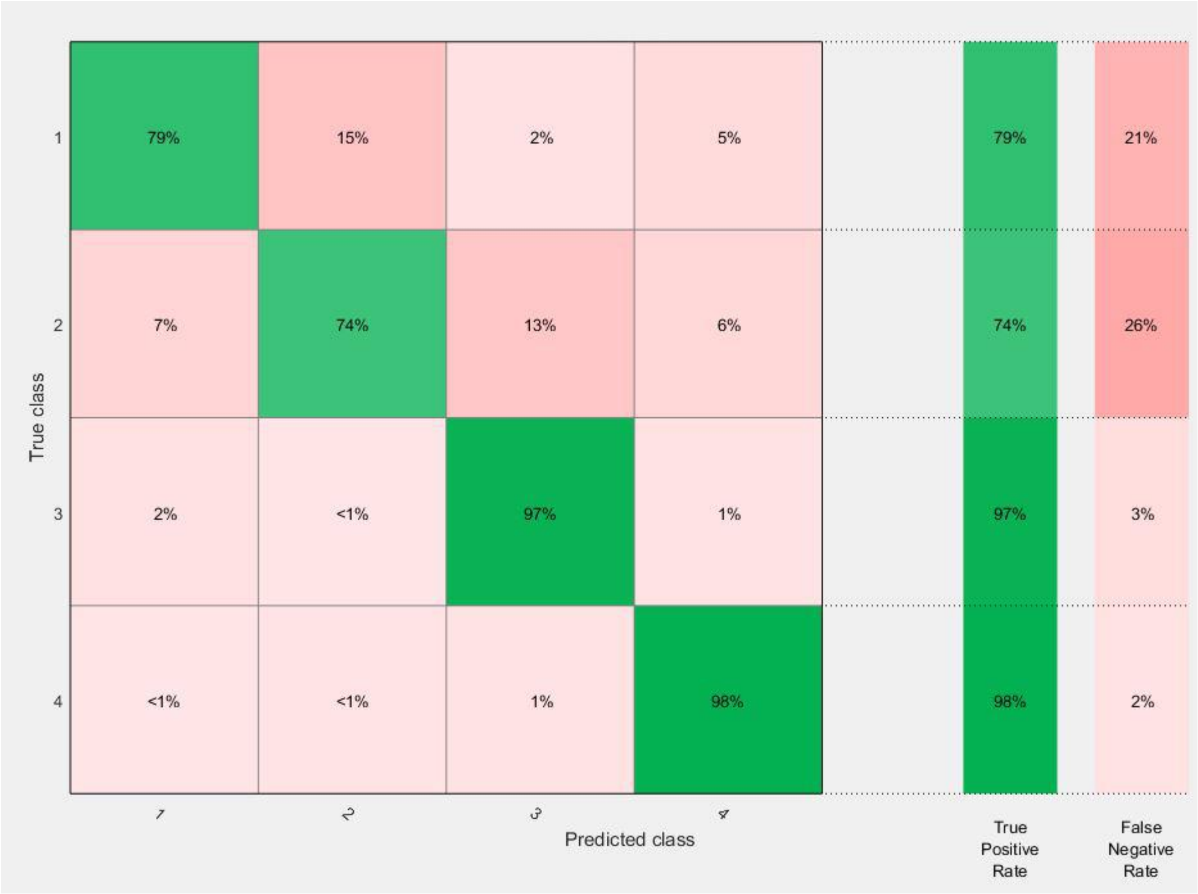

Figure SR4. Confusion matrix with participant's emotional ratings as predictor variable ("True Class") and the true emotional categories as outcome ("Predicted class"). The numbers in the y- and x- axes indicate the different emotional categories (1 = Anger; 2 = Happy; 3 = Neutral; 4 = Fear).

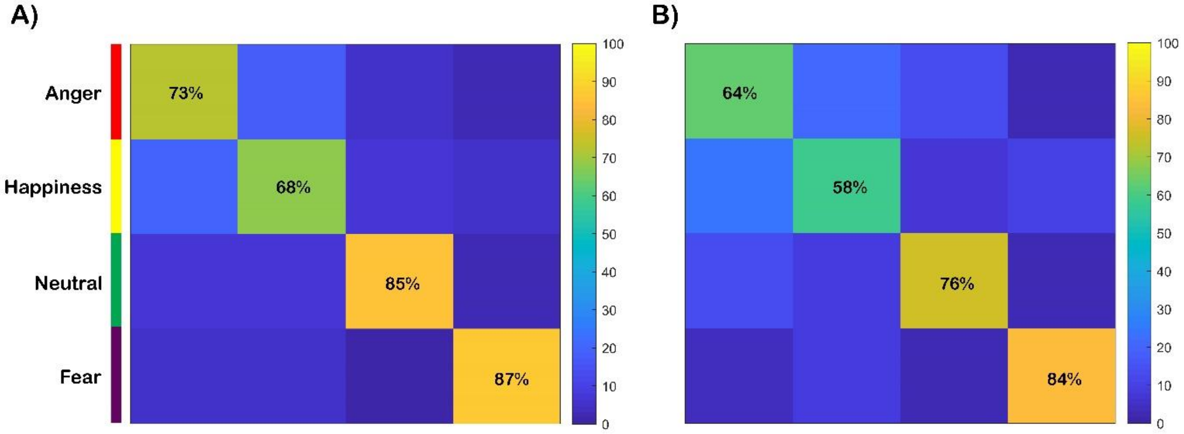

Figure SR5. Confusion matrices showing the classification accuracy by emotional category of the models using behavioural ratings. A) Emotion classification accuracy of the model where the ratings of postural (i.e. contraction and symmetry), kinematic (i.e. amount of movement, fast movement, vertical movement and forward/away) and emotional (i.e. emotional intention, valence and familiarity) traits were included (overall classification accuracy of 78%, with chance level at 25%); **B)** Predictor relevance for the classification model where only postural and kinematic ratings were included (overall classification accuracy of 71%, with chance level at 25%). Colour lines in the left indicate the organization of the matrices with respect to the emotional category (anger: red; happiness: yellow; neutral: green; fear: purple).

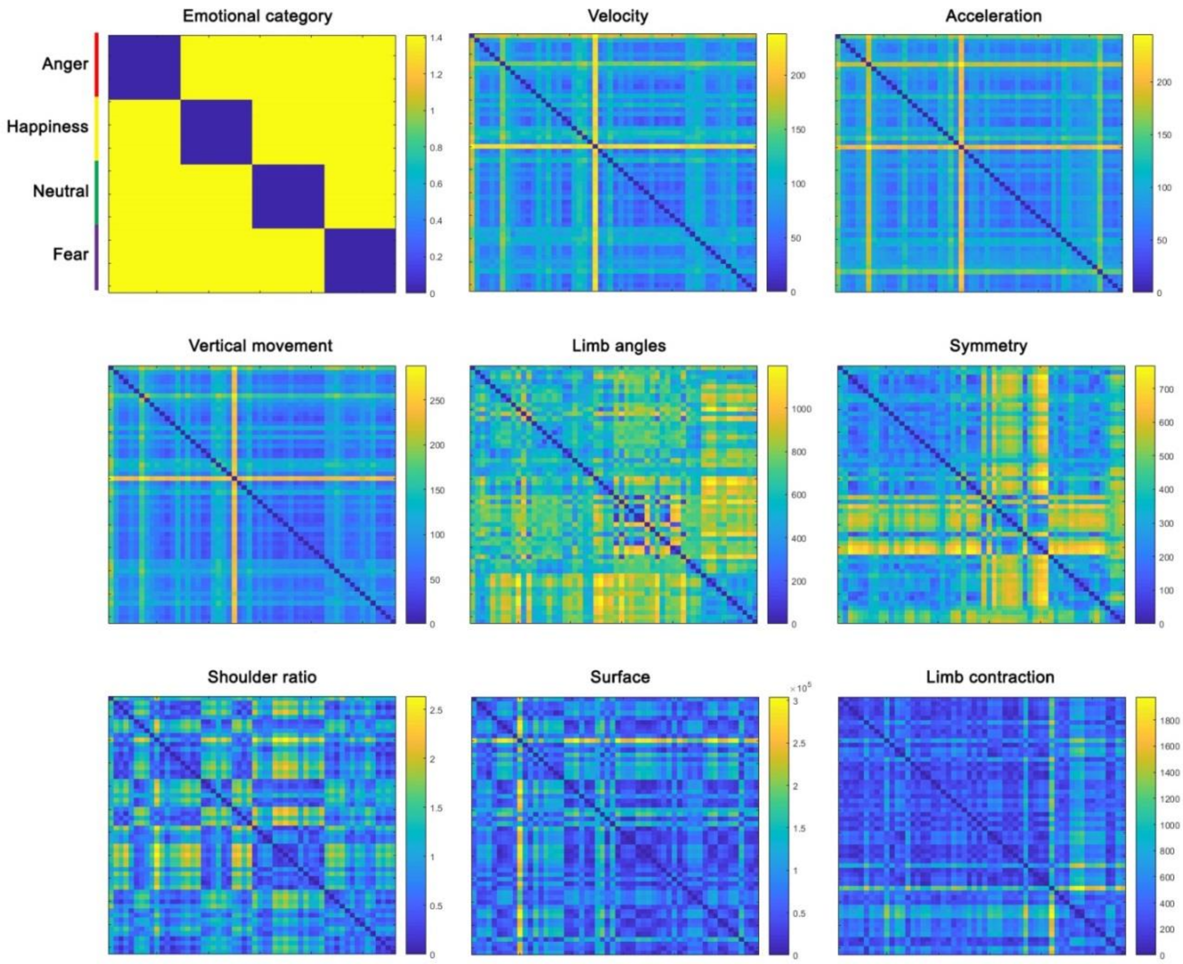

Figure SR6. Representational dissimilarity matrices of the kinematic and postural features preserving the time information. The RDMs represent pairwise comparisons between 56 stimuli with regard to the kinematic and postural computed features. Kinematic features included velocity, acceleration and vertical movement. Postural features included limb angles, symmetry, shoulder ratio, surface and limb contraction. The dissimilarity measure reflects Euclidean distance, with blue indicating strong similarity and yellow strong dissimilarity. Colour lines in the upper left corner indicate the organization of the RDMs with respect to the emotional category (anger: red; happiness: yellow; neutral: green; fear: purple) of the video stimuli.

TABLE SR1. Correlation between representational dissimilarity matrices of kinematic and postural features. Kinematic features included velocity, acceleration and vertical movement. Postural features included limb angles, symmetry, shoulder ratio, surface and limb contraction. The correlations were computed using Spearman's rank correlation. Both uncorrected and Bonferroni-corrected p-values are displayed for each comparison (α_{bonf} = 0.05/9, with nine comparisons per feature).

	**Emotional category**			**Velocity**			**Acceleration**			**Vertical movement**		
	r	p-value	p_{Bonf}-value	r	p-value	p_{Bonf}-value	r	p-value	p_{Bonf}-value	r	p-value	p_{Bonf}-value
**Velocity**	.094	.000	.002									
**Acceleration**	.101	.000	.001	.768	.000	.000						
**Vertical movement**	.057	.025	.227	.417	.000	.000	.120	.000	.000			
**Limb angles**	.251	.000	.000	-.096	.000	.002	-.032	.210	.999	-.208	.000	.000
**Symmetry**	.262	.000	.000	-.054	.033	.294	-.009	.721	.999	-.155	.000	.000
**Shoulder ratio**	.185	.000	.000	.094	.000	.002	.051	.045	.404	.041	.111	.998
**Surface**	.036	.161	.999	.026	.305	.999	-.016	.543	.999	.087	.001	.006
**Limb contraction**	.112	.000	.000	-.104	.000	.000	.033	.200	.999	-.115	.000	.000

TABLE SR1 (continuation). Correlation between representational dissimilarity matrices of kinematic and postural features. Kinematic features included velocity, acceleration and vertical movement. Postural features included limb angles, symmetry, shoulder ratio, surface and limb contraction. The correlations were computed using Spearman's rank correlation. Both uncorrected and Bonferroni-corrected p-values are displayed for each comparison (α_{bonf} = 0.05/9, with nine comparisons per feature).

	**Limb angles**			**Symmetry**			**Shoulder ratio**			**Surface**		
	r	p-value	p_{Bonf}-value	r	p-value	p_{Bonf}-value	r	p-value	p_{Bonf}-value	r	p-value	p_{Bonf}-value
**Symmetry**	.377	.000	.000									
**Shoulder ratio**	.274	.000	.000	.119	.000	.000						
**Surface**	.136	.000	.000	-.057	.026	.232	.547	.000	.000			
**Limb contraction**	.261	.000	.000	.029	.257	.999	-.019	.452	.999	.045	.077	.691

Table SR2. The interclass correlation coefficient (ICC) was used to assess the inter-rater agreement for all the behavioral ratings. An ICC was computed per behavioural rating in SPSS using Absolute-Agreement and a 2-Way Random-Effects Model.

	Mean	Standard deviation	ICC	CI
**Emotional rating**	2.61	0.09	0.99	(0.99, 1.00)
**Emotional intensity**	4.65	0.48	0.98	(0.97, 0.99)
**Valence**	3.58	0.43	0.98	(0.97, 0.99)
**Action-category rating**	2.61	0.09	0.98	(0.98, 0.99)
**Familiarity**	4.50	0.76	0.90	(0.85, 0.93)
**Contraction**	3.80	0.67	0.95	(0.93, 0.97)
**Symmetry**	3.76	0.63	0.95	(0.93, 0.97)
**Amount of movement**	4.15	0.58	0.98	(0.97, 0.99)
**Fast movement**	3.82	0.54	0.97	(0.96, 0.98)
**Vertical movement**	3.27	0.53	0.96	(0.94, 0.97)
**Forward/away**	4.10	0.24	0.99	(0.98, 0.99)

Abbreviations: ICC: interclass correlation coefficient; CI: confidence intervals.

Table SR3. Correlation between representational dissimilarity matrices of the behavioural ratings. The correlations were computed using Spearman's rank correlation. Both uncorrected and Bonferroni-corrected p-values are displayed for each comparison (α_{bonf} =0.05/12, with 12 comparisons per behavioural rating).

	**Emotional Categories**			**Emotional Rating**			**Emotional Intensity**			**Valence**		
	r	p-value	p_{Bonf}-value	r	p-value	p_{Bonf}-value	r	p-value	p_{Bonf}-value	r	p-value	p_{Bonf}-value
**Emotional rating**	.728	.000	.000									
**Emotional intensity**	.414	.000	.000	.455	.000	.000						
**Valence**	.466	.000	.000	.580	.000	.000	.246	.000	.000			
**Action rating**	.720	.000	.000	.887	.000	.000	.466	.000	.000	.602	.000	.000
**Familiarity**	.337	.000	.000	.181	.000	.000	.415	.000	.000	.323	.000	.000
**Contraction**	.414	.000	.000	.474	.000	.000	.576	.000	.000	.343	.000	.000
**Symmetry**	.268	.000	.000	.311	.000	.000	.389	.000	.000	.279	.000	.000
**Amount of movement**	.323	.000	.000	.394	.000	.000	.712	.000	.000	.207	.000	.000
**Fast movement**	.270	.000	.000	.370	.000	.000	.599	.000	.000	.229	.000	.000
**Vertical movement**	.213	.000	.000	.251	.000	.000	.257	.000	.000	.363	.000	.000
**Forward-away**	.535	.000	.000	.477	.000	.000	.018	.468	.999	.147	.000	.000

Table SR3 (continuation). Correlation between representational dissimilarity matrices of the behavioural ratings. The correlations were computed using Spearman's rank correlation. Both uncorrected and Bonferroni-corrected p-values are displayed for each comparison (α_{bonf} =0.05/12, with 12 comparisons per behavioural rating).

	Action Rating			Familiarity			Contraction			Symmetry		
	r	p-value	p_{Bonf}-value	r	p-value	p_{Bonf}-value	r	p-value	p_{Bonf}-value	r	p-value	p_{Bonf}-value
**Familiarity**	.216	.000	.000									
**Contraction**	.477	.000	.000	.345	.000	.000						
**Symmetry**	.340	.000	.000	.234	.000	.000	.252	.000	.000			
**Amount of movement**	.396	.000	.000	.245	.000	.000	.484	.000	.000	.337	.000	.000
**Fast movement**	.347	.000	.000	.155	.000	.000	.425	.000	.000	.288	.000	.000
**Vertical movement**	.269	.000	.000	.121	.000	.000	.289	.000	.000	.188	.000	.000
**Forward-away**	.441	.000	.000	.031	.219	.999	.158	.000	.000	-.020	.428	.999

Table SR3 (continuation). Correlation between representational dissimilarity matrices of the behavioural ratings. The correlations were computed using Spearman's rank correlation. Both uncorrected and Bonferroni-corrected p-values are displayed for each comparison (α_{bonf} =0.05/12, with 12 comparisons per behavioural rating).

	Amount of movement			Fast movement			Vertical movement		
	r	p-value	p_{Bonf}-value	r	p-value	p_{Bonf}-value	r	p-value	p_{Bonf}-value
**Fast movement**	.797	.000	.000						
**Vertical movement**	.472	.000	.000	.487	.000	.000			
**Forward-away**	.044	.086	.999	.003	.916	.999	-.014	.589	.999

Table SR4. Correlation between representational dissimilarity matrices of the behavioural ratings and the computed features. The correlations were computed using Spearman's rank correlation. Both uncorrected and Bonferroni-corrected p-values are displayed for each comparison (α_{bonf} =0.05/12, with 12 comparisons per behavioural rating).

	Computed features														
	Emotional category			Velocity			Acceleration			Vertical movement			Limb angles		
Behavioural ratings	r	p-value	p_{Bonf}-value	r	p-value	p_{Bonf}-value	r	p-value	p_{Bonf}-value	r	p-value	p_{Bonf}-value	r	p-value	p_{Bonf}-value
**Emotional category**	1.000	.000	.000	.094	.000	.003	.101	.000	.001	.057	.025	.302	.251	.000	.000
**Emotional rating**	.728	.000	.000	.174	.000	.000	.147	.000	.000	.053	.037	.445	.280	.000	.000
**Emotional intensity**	.414	.000	.000	.100	.000	.001	.150	.000	.000	-.079	.002	.023	.251	.000	.000
**Valence**	.466	.000	.000	.226	.000	.000	.112	.000	.000	0134	.000	.000	.121	.000	.000
**Action rating**	.720	.000	.000	.123	.000	.000	.076	.003	.032	.065	.010	.123	.339	.000	.000
**Familiarity**	.337	.000	.000	.018	.479	.999	.086	.001	.009	-.107	.000	.000	.133	.000	.000
**Contraction**	.414	.000	.000	.075	.003	.038	.152	.000	.000	-.061	.016	.191	.325	.000	.000
**Symmetry**	.268	.000	.000	.109	.000	.000	.063	.013	.158	-.019	.446	.999	.242	.000	.000
**Amount of movement**	.323	.000	.000	.340	.000	.000	.319	.000	.000	.015	.561	.999	.202	.000	.000
**Fast movement**	.270	.000	.000	.441	.000	.000	.423	.000	.000	.026	.311	.999	.109	.000	.000
**Vertical movement**	.213	.000	.000	.555	.000	.000	.334	.000	.000	.328	.000	.000	.010	.692	.999
**Forward-away**	.535	.000	.000	.035	.170	.999	.077	.003	.031	.147	.000	.000	.160	.000	.000

Table SR4 (continuation). Correlation between representational dissimilarity matrices of the behavioural ratings and the computed features. The correlations were computed using Spearman's rank correlation. Both uncorrected and Bonferroni-corrected p-values are displayed for each comparison (α_{bonf} =0.05/12, with 12 comparisons per behavioural rating).

	Computed features											
	Symmetry			Shoulder ratio			Surface			Limb contraction		
Behavioural ratings	r	p-value	p_{Bonf}-value	r	p-value	p_{Bonf}-value	r	p-value	p_{Bonf}-value	r	p-value	p_{Bonf}-value
**Emotional category**	.262	.000	.000	.185	.000	.000	.036	.161	.999	.112	.000	.000
**Emotional rating**	.300	.000	.000	.220	.000	.000	.075	.003	.039	.119	.000	.000
**Emotional intensity**	.400	.000	.000	.356	.000	.000	-.024	.343	.999	.113	.000	.000
**Valence**	.049	.057	.684	.279	.000	.000	.218	.000	.000	.003	.915	.999
**Action rating**	.365	.000	.000	.296	.000	.000	.136	.000	.000	.123	.000	.000
**Familiarity**	.155	.000	.000	.207	.000	.000	-.018	.470	.999	.103	.000	.001
**Contraction**	.332	.000	.000	.147	.000	.000	.005	.853	.999	.329	.000	.000
**Symmetry**	.366	.000	.000	.330	.000	.000	.178	.000	.000	.049	.054	.648
**Amount of movement**	.343	.000	.000	.233	.000	.000	-.014	.589	.999	.109	.000	.000
**Fast movement**	.224	.000	.000	.157	.000	.000	-.048	.061	.736	.067	.009	.108
**Vertical movement**	.013	.602	.999	.136	.000	.000	.028	.264	.999	-.021	.420	.999
**Forward-away**	.096	.000	.002	.000	.997	.999	-.027	.284	.999	.180	.000	.000

References

1 Kleinsmith, A. & Bianchi-Berthouze, N. Affective body expression perception and recognition: A survey. *IEEE Trans. Affect. Comput.* **4**, 15-33 (2012).

2 Cao, Z., Simon, T., Wei, S.-E. & Sheikh, Y. Realtime multi-person 2d pose estimation using part affinity fields. *Proceedings of the IEEE Conference on Computer Vision and Pattern Recognition*, 7291-7299 (2017).
